# Metformin can mitigate skeletal dysplasia caused by *Pck2* deficiency

**DOI:** 10.1038/s41368-022-00204-1

**Published:** 2022-11-15

**Authors:** Zheng Li, Muxin Yue, Boon Chin Heng, Yunsong Liu, Ping Zhang, Yongsheng Zhou

**Affiliations:** 1 Department of Prosthodontics, Peking University School and Hospital of Stomatology, Beijing, China; 2 National Center of Stomatology & National Clinical Research Center for Oral Diseases & National Engineering Research Center of Oral Biomaterials and Digital Medical Devices & Beijing Key Laboratory of Digital Stomatology, 22 Zhongguancun South Avenue, Haidian District, Beijing, China; 3 The Central Laboratory, Peking University School and Hospital of Stomatology, Beijing, China

**Keywords:** Bone development, Differentiation

## Abstract

As an important enzyme for gluconeogenesis, mitochondrial phosphoenolpyruvate carboxykinase (PCK2) has further complex functions beyond regulation of glucose metabolism. Here, we report that conditional knockout of *Pck2* in osteoblasts results in a pathological phenotype manifested as craniofacial malformation, long bone loss, and marrow adipocyte accumulation. Ablation of *Pck2* alters the metabolic pathways of developing bone, particularly fatty acid metabolism. However, metformin treatment can mitigate skeletal dysplasia of embryonic and postnatal heterozygous knockout mice, at least partly via the AMPK signaling pathway. Collectively, these data illustrate that PCK2 is pivotal for bone development and metabolic homeostasis, and suggest that regulation of metformin-mediated signaling could provide a novel and practical strategy for treating metabolic skeletal dysfunction.

## Introduction

Craniofacial bone formation is critical for brain, face, and oral development and functions. Developmental abnormalities often alter craniofacial structures resulting in craniofacial skeletal dysplasia, such as mandibular hypoplasia and cleft palate, which severely affect physiological functions.^1^ Besides congenital defects, various systemic diseases can cause severe deformities not only of the long bones but also of the craniofacial bone. For example, Job’s syndrome patients are highly susceptible to long bone fractures, osteopenia and craniosynostosis,^2,3^ while Ellis-van Creveld syndrome (EvC) patients often exhibit mandibular prognathism and skull enlargement.^4^ Although numerous clinical studies have attempted to explore the underlying molecular mechanisms of craniofacial bone abnormities, the current understanding of their pathogenesis and clinical treatment efficacy remains limited.

The skeletal system is regulated by complex metabolic networks that are precisely tuned to maintain bone homeostasis. Dysregulation of these metabolic processes often results in multiple congenital or postnatal skeletal disorders. Metabolic pathways are constituted of a series of complex enzymatic cascades and glucose metabolism is one of the important links.^5^ As a critical rate-limiting enzyme in gluconeogenesis, phosphoenolpyruvate carboxykinase (PCK) has two isoforms, cytosolic isoform (PCK1) and mitochondrial isoform (PCK2).^6^ They are expressed in specific tissues and species at varying proportions. Commonly, PCK1 is highly expressed in kidney, liver, which are exclusively gluconeogenesis organs, while PCK2 displays a broader expression profile that can be induced by cellular or tissue stress.^7^ The metabolic characteristics of PCK1 have been broadly studied, but the functions of PCK2 are still unexplored because PCK1 accounts for at least 95% of total PCK-activity in mouse liver, and mice are the most commonly used transgenic models for in vivo study.^6^ Notably, PCK1 is highly enriched in human adipose tissue while PCK2 is enriched in human bone tissue.^8^ Our previous work had first reported that PCK2 could promote osteogenesis through an autophagy-dependent manner in two-dimensional (2D) culture,^9^ and also further revealed that PCK2 could modulate osteogenesis through glycolysis responding to three-dimensional (3D) extracellular matrix (ECM) stiffness.^10^ However, the in vivo role and the molecular mechanisms of PCK2 in the development of craniofacial and long bones are not clear.

Metformin is a widely used drug for the treatment of type 2 diabetes mellitus (T2DM), but more recently, it has been demonstrated to promote bone formation by stimulating osteogenesis and protecting osteoblasts and mesenchymal stem cells (MSCs) from hyperglycaemia.^11^ Moreover, the pharmacological effect of metformin is mild, safe, and well-tolerated. Accumulating scientific evidence has shown that metformin enhances bone formation due to its metabolic effects on glucose and fatty acid regulation.^12,13^ Considering the widespread use of metformin for treating T2DM and other diseases, we raised the question whether metformin can also play a role in regulating PCK2-mediated bone formation.

In this study, we generated *Pck2* conditional knockout mice and investigated the key roles of PCK2 deficiency in craniofacial bone dysplasia and long bone loss during embryonic and postnatal stages. It was demonstrated that *Pck2* deletion could alter the metabolic profiles of developing bone tissue. Additionally, we showed that metformin could mitigate craniofacial bone deformity and long bone loss, at least partially via AMPK signaling. Our findings thus unveiled the key regulatory roles of *Pck2* in skeletal development, and suggested that metformin could potentially be utilized for the treatment of metabolic bone diseases.

## Results

### Loss of *Pck2* leads to craniofacial skeletal development disorder

**Fig. 1:**
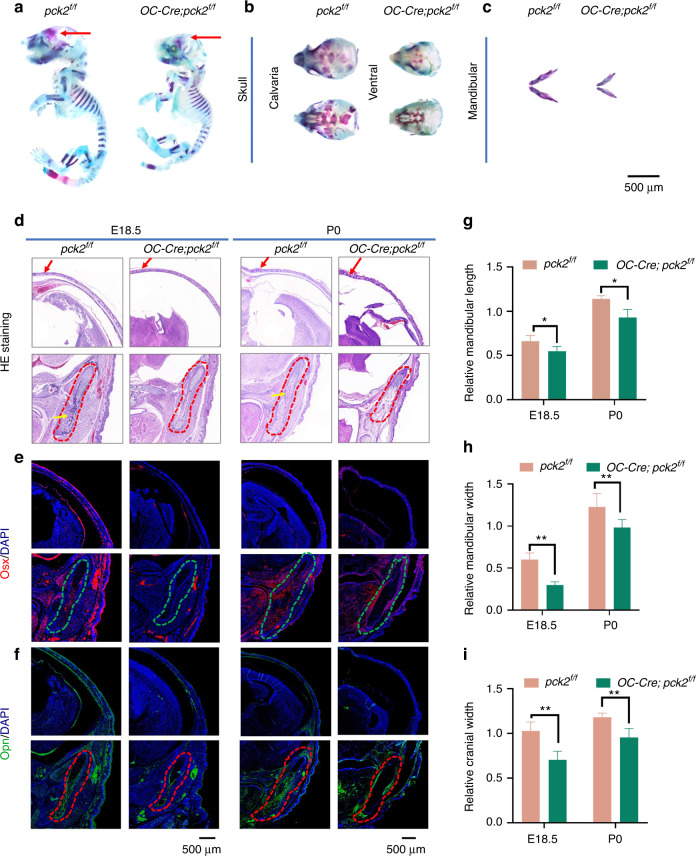
*Pck2* is indispensable for craniofacial skeletal development. **a**–**c** Whole-mount Alizarin red and Alcian blue staining of *Pck2* KO and littermate control mice at E18.5, including skulls (**b**) and mandibles (**c**). Arrows (skull in **a**) indicate that the KO mice displayed less bone formation compared with the control mice. Scale bars: 500 μm. *n* = 3. **d**–**f** Images of HE staining (**d**), *Osx* IF staining (**e**), *Opn* IF staining (**f**) of the head sections from E18.5 embryos or P0 pups with indicated genotypes. Cranial bone width was indicated with arrows in **d**. Scale bars: 500 μm. *n* = 3. **g–i** Quantitative analysis of relative mandibular length (*n* = 3), relative mandibular width (*n* = 3), and relative cranial width (*n* = 3). **P < 0.01; *P < 0.05

To investigate whether PCK2 affects craniofacial skeletal development, we constructed an osteoblast-conditional *Pck2* knockout mouse model by crossing *Pck2*^*f/f*^ mice with *Osteocalcin-Cre* (*OC-Cre*) mice (*OC-Cre; Pck2*^*f/f*^), using the CRISPR/Cas9 system (Fig. S1a). In this mouse line, Cre expression is restricted to osteoblast, and the *OC-Cre* transgene is intended to target the mature osteoblast lineage, including endosteum, periosteum, and osteocytes.^14^ The comparison was between *Pck2*^*f/f*^ (control group) and *OC-Cre;* versus *Pck2*^*f/f*^ (experimental group), and the *Pck2* knockout efficiency was confirmed by western blot and quantitative reverse transcriptase polymerase chain reactions (qRT-PCR) (Fig. S1b). The whole body Alcian blue and Alizarin red S staining showed that the physical size of E18.5 *OC-Cre; Pck2*^*f/f*^ mice were relatively smaller than their control littermates, particularly the cranial and mandibular bones (Fig. 1a–c). The cranial and mandibular bones of E18.5 *OC-Cre; Pck2*^*f/f*^ mice displayed hypo-mineralized properties compared with the control mice (Fig. 1b, c). Histological analysis of the frontal sections of the heads of *OC-Cre; Pck2*^*f/f*^ mice showed smaller cranial bones compared with the *Pck2*^*f/f*^ mice at E 18.5 and P0 (Fig. 1d). In addition, the mandibular length and width were significantly reduced in conditional *Pck2* knockout mice, in both embryos and neonates. The control group had a more homogeneous mandibular osteoid structure with a few embedded blood vessels (yellow arrows), while the *OC-Cre; Pck2*^*f/f*^ groups displayed a sparser bone-like structure without any significant angiogenesis (Fig. 1d). *Osterix (Osx)* is an important transcription factor for early osteogenesis,^2^ and it was found to be downregulated in *OC-Cre; Pck2*^*f/f*^ embryos and newborns. *Osx* positive cells were decreased in the cranial and mandibular bones of *OC-Cre; Pck2*^*f/f*^ compared with the *Pck2*^*f/f*^ mice both at E18.5 and P0 (Fig. 1e). Additionally, the marker for mature osteoblasts- *Osteopontin (Opn)*,^15^ was downregulated at E18.5 and P0 in the jaw and calvaria of the *OC-Cre; Pck2*^*f/f*^ mice, as compared with *Pck2*^*f/f*^ mates (Fig. 1f). *Osx*-positive and *Opn*-stained areas outlined the calvaria and mandibular bone, and we have assessed the size of the jaw and the thickness of the cranial bone. Compared with control mice, the mandibular size (length and width) and the cranial bone thickness of *OC-Cre; Pck2*^*f/f*^ mice were relatively reduced both at E18.5 and P0, but this phenotype was more obvious at E18.5, suggesting the key role of *Pck2* in the early embryo development of craniofacial bone (Fig. 1g–i).

### Deletion of Pck2 induces bone loss in the developing long bone

**Fig. 2:**
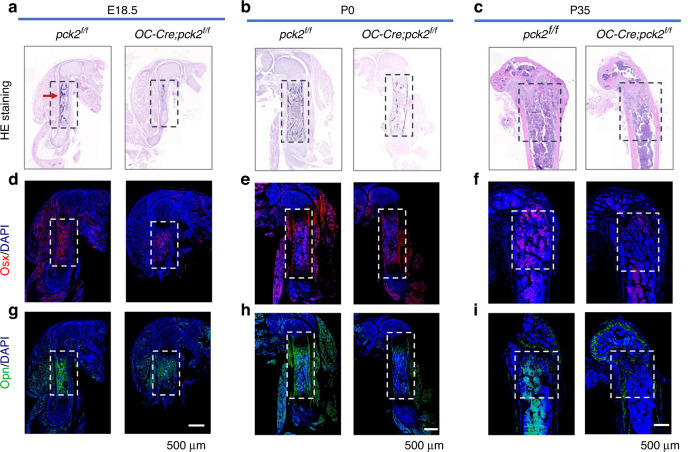
*Pck2* is necessary for long bone development. **a**–**c** HE staining of the femur sections from E18.5 (**a**) or P0 (**b**) or P35 (**c**) with indicated genotypes. Scale bars: 500 μm. *n* = 3. Arrows in **a** indicated the micro-vessel-like structure in the femur of E18.5 *Pck2*^*f/f*^ mice. **d**–**i** Representative images of *Osx* IF staining (**d**–**f**), *Opn* IF staining (**g**–**i**) of the femur sections from E18.5 (**d**, **g**) or P0 (**e**, **h**) or P35 (**f**, **i**) with indicated genotypes. Scale bars: 500 μm. *n* = 3

Since *Pck2* exerted a critical role in craniofacial bone development, we, therefore, investigated whether *Pck2* loss could alter long bone mass. We analyzed the femurs of E18.5, P0 and P35 *OC-Cre; Pck2*^*f/f*^ mice and their control littermates. Histological analysis showed that the *Pck2* deletion resulted in no significant change in femur size at P0 and P35, but at E18.5, the femur was slightly smaller. As shown by HE staining (Fig. 2a), the *OC-Cre; Pck2*^*f/f*^ embryo pups showed less immature bone-like and vessel-like structures, compared with their control littermates. In the newborn and adult femurs, the trabecular was not evenly distributed and the staining of the trabecular was not that deep in *OC-Cre; Pck2*^*f/f*^ mice (Fig. 2b, c). The quantification of osteoid area in femurs at E18.5 and P0 and the percentage of trabecular in femurs at P35 were shown in Fig. S1c. Developing long bone sections of E18.5, P0 and P35 were subjected to immunofluorescence (IF) staining to analyze the cellular mechanisms of skeletal dysplasia. Consistently, the ablation of *Pck2* in osteoblast led to reduced *Osx* and *Opn* expression in the embryonic, newborn, and adult *OC-Cre; Pck2*^*f/f*^ mice, as compared with *Pck2*^*f/f*^ mice (Fig. 2d–i). Downregulation of *Col1a1*, *Osx*, *Runx2*, and *Alp* in the developing long bones of the E18.5 *OC-Cre; Pck2*^*f/f*^ mice was further confirmed by qPCR (Fig. S1d).

**Fig. 3:**
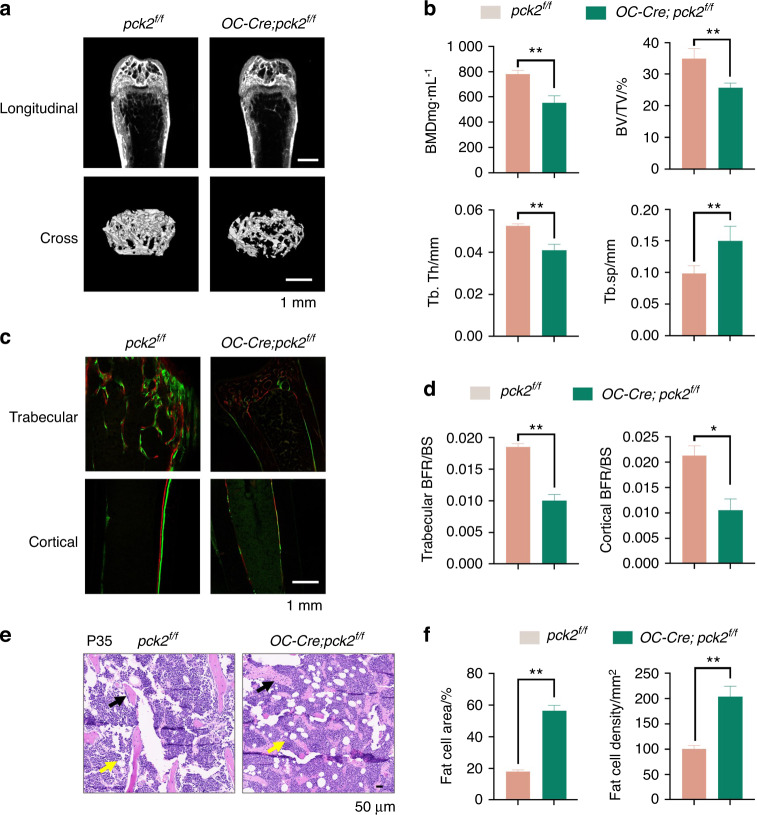
*Pck2* deficiency in osteoblasts caused decreased long bone mass and accumulated marrow adiposity. **a** Representative images of femur micro-CT from P35 mice with indicated genotypes. Scale bars, 1 mm, *n* ≥ 3. **b** Quantitative analysis of micro-CT scanning, including BMD, BV/TV%, Tb.Th, Tb.Sp. **P < 0.01, *n* ≥ 3. **c** Images of femur calcein-alizarin red S double labeling from P35 mice with indicated genotypes. **d** Analysis of trabecular and cortical bone formation rate (BFR). **P < 0.01, *P < 0.05, *n* = 3. **e** Images of adipose in the femur distal femur marrow in *OC-Cre; Pck2*^*f/f*^ mice and *Pck2*^*f/f*^ mice at P35. Black arrows present trabecular bones, and yellow arrows point to marrow adipose tissues. Scale bar, 50 μm, *n* = 3. **f** Quantification of adipocytes, including number and adipocyte area per tissue area in the femur distal marrow, which were analyzed with the ImageJ software. **P < 0.01, *n* = 3

Then we measured the bone mass of the adult (P35) distal femur metaphysis by micro-computed tomography (Micro-CT) analysis (Fig. 3a, b). The results showed a greater reduction of bone mineral density (BMD) and bone volume/tissue volume ratio (BV/TV) in *OC-Cre; Pck2*^*f/f*^ mice. Moreover, *Pck2* mutant mice displayed significantly decreased trabecular thickness (Tb. Th) and incremental trabecular spacing (Tb. Sp) than the control littermates at P35 (Fig. 3a, b). To examine whether the bone loss was caused by impaired bone formation, we conducted a dynamic histomorphometric analysis using calcein and alizarin red labeling. An obvious decrease in calcein and alizarin labeling was shown in the bone of the *OC-Cre; Pck2*^*f/f*^ mice, and the bone formation rate (BFR) of trabecular and cortical bone in *OC-Cre; Pck2*^*f/f*^ mice were significantly downregulated compared with *Pck2*^*f/f*^ mice (Fig. 3c, d) at P35. Notably, bone loss caused by *Pck2* deletion was accompanied by obvious accumulation of bone marrow adipose tissue (MAT) (Fig. 3e, Fig. S1e) and increased density and number of adipocytes in the bone marrow at P35 (Fig. 3f). These results suggested that *Pck2* played a critical role in skeletal formation from embryonic to postnatal life.

### Pck2 ablation alters metabolic profile in the developing bone

**Fig. 4:**
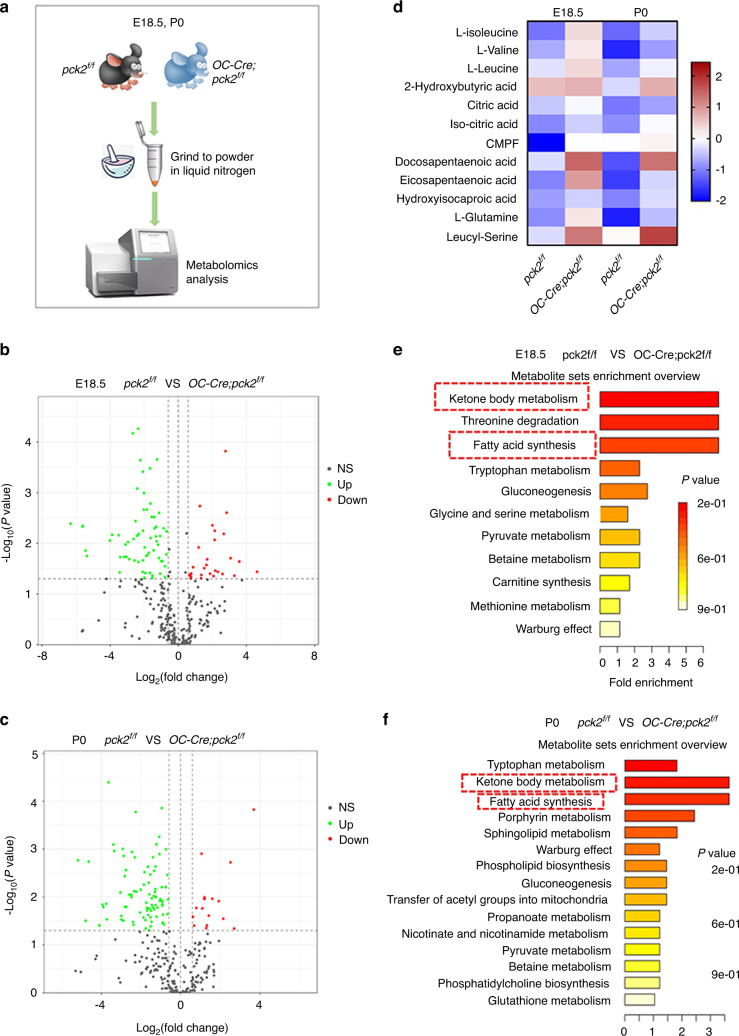
*Pck2* ablation in osteoblasts brought about changes in the metabolic profiles of developing long bones. **a** Scheme illustration of the process for isolating E18.5 and P0 pups for metabolic analysis, *n* = 3. **b**, **c** Volcano plot showing both the statistical significance (*P* value) and the magnitude of change (fold change) of differentially expressed metabolites in the KO and control mice at E18.5 and P0. **d** Heatmap analysis of representative differentially expressed metabolites between control and *OC-Cre; Pck2*^*f/f*^ KO Samples. **e**, **f** Metabolite set enrichment overview in *OC-Cre; Pck2*^*f/f*^ mice and *Pck2*^*f/f*^ mice at E18.5 (**e**) and P0 (**f**). The *P* value was less than 0.05

As a key enzyme of gluconeogenesis, *Pck2* has been shown to regulate metabolic reprogramming.^16^ In order to characterize the metabolic profile changes upon *Pck2* deletion, and thus explore the underlying mechanisms by which *Pck2* regulate osteoblast lineage commitment, we performed precise untargeted metabolomics to broadly identify metabolic pathways altered by *Pck2* loss. The limbs from the E18.5 embryos and P0 newborns were isolated, and both of the end of limbs including cartilage tissues were removed, in order to exclude the influence of cartilage differentiation (Fig. 4a). Due to the great energetic demand to maintain bone homeostasis, all the cells in the skeletal system are closely interacted with each other through neuronal and hormonal molecules.^5^ When the metabolic microenvironment changes, the metabolic condition of heterogeneous populations in bone marrow including stromal cells, endothelial cells, and some hematopoietic cells changes correspondingly.^17,18^ Therefore, we chose to measure the whole bone marrow lysates (including bone marrow and cortical bone) for metabolic analysis. The experiment was conducted in triplicate, and significant metabolic changes were revealed after ablation of *Pck2* at E18.5 and P0, giving the solid hint that bone is an organic coordinated system and bone metabolism is an interlocking process in which multiple cells work together as a whole, and *Pck2* deletion in osteoblast led to the metabolic changes in skeletal system. A panel of various metabolites were captured, which were the main elements of the TCA cycle, fatty acid metabolism, and amino acid metabolism. The overall metabolite sets enriched by differential metabolites between the *OC-Cre; Pck2*^*f/f*^ mice and *Pck2*^*f/f*^ mice at E18.5 and P0, are shown in Fig. 4b, c. The heatmap based on hierarchical clustering analysis (Fig. 4d) illustrated that in the bone tissue from *OC-Cre; Pck2*^*f/f*^ mice, L-isoleucine, L-leucine and L-valine, which could be classified as branched-chain amino acids (BCAAs) were upregulated, compared with their littermate controls. Ketone bodies are the intermediate products of the oxidative breakdown of fatty acids in the liver and the 2-hydroxybutyric acid is the main product.^19^ The 2-Hydroxybutyric acid was accumulated due to *Pck2* loss, both in embryos and newborns (Fig. 4d). Ketone bodies are the breakdown products of fat, not glucose.^20^ Unexpectedly, we found that the accumulation of fatty acid increased in *OC-Cre; Pck2*^*f/f*^ mice, including 3-carboxy-4-methyl-5-pentyl-2-furanpropanoic acid (CMPF), docosapentaenoic acid, eicosapentaenoic acid, and hydroxyisocaproic acid. Acetyl CoA carboxylase is the rate-limiting enzyme of fatty acid synthesis, which is present within the cytosol (Fig. 4d). Citric acid and isocitric acid are the metabolic activators of Acetyl CoA carboxylase. Therefore, citric acid could allosterically activate this enzyme to promote fatty acid synthesis.^21^ As shown in Fig. 4d, we demonstrated that the levels of citric acid and iso-citric acid were increased due to deletion of *Pck2*. PCK2 was reported to accelerate oxaloacetate-derived carbons into *de novo* synthesized serine.^22^ Our results revealed that leucyl-serine was downregulated in the *OC-Cre; Pck2*^*f/f*^ mice, both at E18.5 and P0. The metabolite sets enriched by differential production of metabolites between *OC-Cre; Pck2*^*f/f*^ mice and *Pck2*^*f/f*^ mice at E18.5 and P0 were outlined by the Metabolite Set Enrichment Analysis (MSEA) (Fig. 4e, f). Within the bone tissues of embryos and newborns, the common enriched metabolic pathways were ketone body metabolism, fatty acid synthesis, pyruvate metabolism, betaine metabolism, Warburg effect, and gluconeogenesis metabolism. Besides metabolites, MSEA showed that threonine degradation, amino acid metabolism (tryptophan metabolism, glycine, and serine metabolism), carnitine synthesis and methionine metabolism were enriched in the bone tissues of E18.5 (Fig. 4e); while in the bone tissues of P0, porphyrin metabolism, phospholipid biosynthesis, propanoate metabolism, nicotinate, and nicotinamide metabolism, transfer of acetyl groups into mitochondria, phosphatidylcholine biosynthesis and glutathione metabolism were enriched (Fig. 4f). These results thus depicted a complicated alteration of metabolic network regulation upon *Pck2* deletion, which in turn prompted us to explore its underlying mechanism and clinical relevance.

### Metformin mitigates skeletal dysplasia of *Pck2*-deficient mice at the embryonic stage

**Fig. 5:**
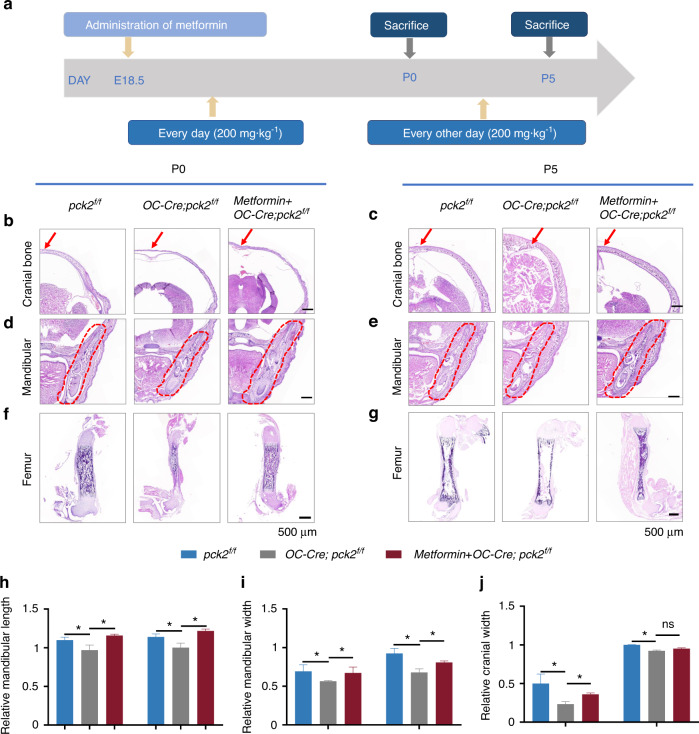
Skeleton dysplasia in *OC-Cre; Pck2*^*f/f*^ mice were mitigated by metformin treatment. **a** The timetable of metformin administration for pregnant mice of indicated phenotypes. **b**–**e** HE staining of the head sections from P0 (**b**, **d**), P5 (**c**, **e**) mice with indicated phenotypes. Cranial bone width was indicated with arrows in **b**, **c**. Scale bars: 500 µm. *n* = 3. **f**, **g** HE staining of the femur sections from P0 (**f**), P5 (**g**) mice with indicated phenotypes. Scale bars: 500 µm. *n* = 3. **h**–**j** Quantitative analysis of relative mandibular length (*n* = 3), relative mandibular width (*n* = 3), and relative cranial width (*n* = 3). *P < 0.05, ns not significant

We found that *Pck2* deletion led to aberrant metabolic profiles of BCCAs, ketone body metabolism and fatty acid synthesis in the developing bone, thus revealing that *Pck2*-mediated signaling is more complicated than gluconeogenesis. Elevated circulating levels of BCAAs, ketone body and free fatty acids are detrimental to cells and tissues.^23^ Besides its fundamental effect on disrupting fatty acid synthesis, metformin was reported to rescue insulin-resistant mice by decreasing circulating BCCAs.^24^ To determine whether metformin exerts any in vivo effect on the function of *Pck2* in developing bone formation, we decided to treat the pregnant mice with metformin (200 mg·kg^−1^) from E18.5, based on the administration timetable as shown in Fig. 5a. This dose is equivalent to an adult dose of 1 000 mg per d at 60 kg, according to the food and drug administration (FDA), which is the starting dose of metformin in humans (500 mg per d twice daily).^25^ Intraperitoneal administration of metformin partially mitigated osteopenia and restored bone formation in the *OC-Cre; Pck2*^*f/f*^ mice. Metformin administration increased the length and width of the mandibles at P0 and P5, as well as the width of cranial bone at P0 (Fig. 5b, d, e, h, i, j). However, the cranial bone width of *OC-Cre; Pck2*^*f/f*^ mice seemed not to be increased after metformin treatment at P5 (Fig. 5c, j). It has been reported that tissue mineral density and development pattern in the calvaria and mandible is different during perinatal development, the possible reason may be attributed to the alteration in mechanical and chemical environment, ossification mode and embryological origin.^26^ The mandibular biomineralization was increased in perinatal and the early newborn stage because of increasing demand to resist masticatory stresses. Calvaria biomineralization increasement may be due to ongoing brain expansion. A possible reason for the failure of metformin to rescue cranial width is that the age-related trajectories throughout the craniofacial bone are affected by the functional specific chemical or physical niches, as well as ontogenetic processes influencing each region.^27^ However, our understanding of cranial bone formation at different regions during early development still needs to be further improved.^28^ Our results showed that metformin also promoted bone formation of *OC-Cre; Pck2*^*f/f*^ mice femurs at P0 and P5, as assessed by the HE staining (Fig. 5f, g). Although not all the bone formation parameters elevated, we could still conclude that metformin partially mitigated skeletal dysplasia of *OC-Cre; Pck2*^*f/f*^ mice at the embryonic stage.

### Metformin mitigates impaired bone loss phenotype in the adult *Pck2* KO mice

**Fig. 6:**
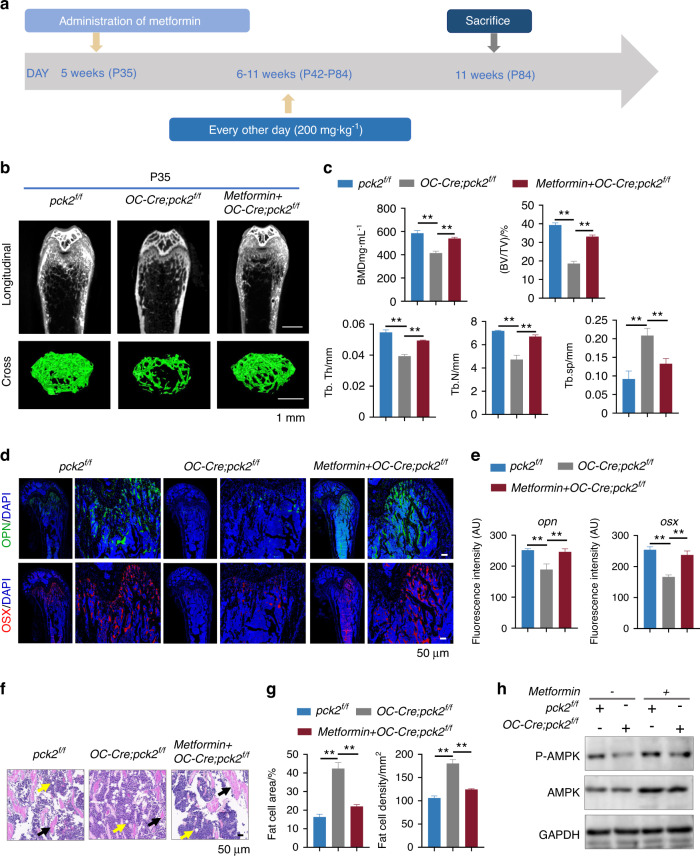
Metformin mitigated the bone loss of the *OC-Cre; Pck2*^*f/f*^ mice. **a** Scheme of metformin administration plan. **b** Representative femur micro-CT images from P35 mice of indicated genotypes. Scale bars, 1 mm. *n* ≥ 3. **c** Quantification of micro-CT parameters in femur bones from P35 mice of indicated genotypes. **P < 0.01. *n* ≥ 3. **d** Representative IF staining images of *Opn* and *Osx* in the femur sections of P35 with indicated genotypes. Scale bars 50 μm. *n* ≥ 3. **e** Quantitative analysis of IF staining of *Opn* and *Osx* in the femur sections of P35 with indicated genotypes. **P < 0.01. *n* ≥ 3. **f** Representative adipocyte images of the distal femur marrow in *OC-Cre; Pck2*^*f/f*^ mice and *Pck2*^*f/f*^ mice at P35. Black arrows suggest trabecular bones, and yellow arrows mean marrow adipose tissues. Scale bar 50 μm, *n* = 3. **g** Quantification of adipocytes, including number and adipocyte area per tissue area in the femur distal marrow, which were analyzed with the ImageJ software. **P < 0.01, *n* = 3. **h** Western blotting analyses of p-AMPK and AMPK in the tissue lysates from the bone of P0 pups with indicated genotypes/treatments. The experiment was repeated twice (*n* = 2)

To explore whether such therapeutic effects of metformin could mitigate bone loss caused by *Pck2* knockout in adult mice, we treated P35 mice with metformin via intraperitoneal injection every other day for 6 weeks, and the administration plan is shown in Fig. 6a. The results of micro-CT analysis showed that metformin administration improved the impaired trabecular bone mass caused by *Pck2* loss, as indicated by the elevated parameters of BMD, BV/TV, Tb.Th., trabecular number (Tb.N) and the decreased Tb.Sp (Fig. 6b, c). Therefore, metformin could mitigate skeletal dysplasia at both the embryonic and postnatal stages, so we next explored the underlying mechanisms. At the molecular level, IF staining and fluorescence intensity quantification suggested that metformin treatment significantly increased *Sp7* and *Opn* expression of *OC-Cre; Pck2*^*f/f*^ mice femurs at P35 (Fig. 6d, e). Moreover, *Pck2* deletion significantly promoted MAT accumulation in P35 *OC-Cre; Pck2*^*f/f*^ mice femurs, as illustrated by an increase in fat cell numbers and adipocyte density, while metformin administration resulted in downregulation of MAT accumulation (Fig. 6f, g, Fig. S1f). To further determine the underlying mechanisms of the therapeutic effects of metformin on Pck2 deletion, the bone lysates from P0 pups were isolated from *Pck2*^*f/f*^, *OC-Cre; Pck2*^*f/f*^ and *OC-Cre; Pck2*^*f/f*^ + metformin mice femurs and tibias. The mRNA expression levels of *Runx2, Osx* and *Alp* suggested that metformin could enhance osteogenesis of mesenchymal cells from *Pck2*^*f/f*^ pup lysates, as well as mitigated the reduced osteogenic potential of *OC-Cre; Pck2*^*f/f*^ mesenchymal cells, as demonstrated by qPCR (Fig. S2a). ALP staining suggested the same trend (Fig. S2b). Our previous work had revealed that PCK2 could promote bone formation via 5’ AMP-activated protein kinase (AMPK)-dependent autophagy.^9^ Additionally, previous studies indicated that AMPK is involved in metformin-mediated osteogenesis in osteoblasts and bone marrow mesenchymal stem cells (BMSCs).^29^ Hence, we focused our analysis on the AMPK signaling pathway. The western blot results demonstrated that the phosphorylation expression of AMPK in both metformin groups was upregulated in P0 pups, as compared with their corresponding control group, respectively (Fig. 6h). The above data thus revealed that metformin could positively regulate osteoblast differentiation and partially rescue the impaired skeletal phenotypes of heterozygous conditional *Pck2* deletion mice via AMPK signaling.

## Discussion

Craniofacial deformities are often caused by various congenital defects or diseases, which manifest as abnormalities of the calvaria and mandibular bones. Illustrating the underlying mechanisms of osteogenesis in craniofacial bone development is a key prerequisite to developing new clinical strategies for craniofacial malformations.^30^ Our previous studies have suggested that PCK2 is a key regulator of osteogenesis within both 2D cultures^9^ and 3D ECM.^10^ Osteoblasts produce a matrix that becomes mineralized during bone formation. In this study, we first established OC transgenic mice for conditional knockout of *Pck2* in osteoblast lineage cells. We found that deletion of *Pck2* in osteoblasts led to various pathological manifestations, such as craniofacial dysplasia, osteoporosis of long bone (bone loss and increased accumulation of marrow adipocytes). Notably, we found that the anti-diabetes drug metformin could rescue bone loss and craniofacial malformation caused by *Pck2* ablation via AMPK signaling, thereby indicating a novel strategy to treat bone metabolic dysfunction.

Bone is a dynamic mineralized structure that has key roles in providing mechanical support and protection to the human body, in addition to containing marrow tissues involved in hematopoiesis, and serving as a repository for calcium storage.^31^ Bone tissue has intrinsic metabolic networks that are closely orchestrated to maintain homeostasis. Disturbances in the metabolic state of bone cells often result in deficient bone formation, with aberrant metabolic processes leading to a range of congenital skeletal and metabolic disorders.^32^ For example, obesity could make bones more fragile; possibly due to the increased accumulation of bone marrow adipocytes and decreased osteoblastogenesis.^33^ However, the complicated relationship between obesity and bone metabolism is still unclear. Recently, PCK-mediated pathways have attracted increasing attention, because it is one of the key pathways that connect the TCA cycle with glycolytic metabolites. Aberrant PCK-related reactions may lead to a series of pathological conditions, including obesity and dysregulation of glucose metabolism.^7^ Adipose-specific Pck1 knockout mice lacked glyceroneogenesis, and exhibit a lipodystrophic form of metabolic syndrome,^34,35^ while adipose-specific *Pck1* overexpressing mice exhibit increased glyceroneogenesis and decreased circulating fatty acid.^36,37^ The broad biological functions of PCK1, besides just gluconeogenesis, prompted us to further investigate whether PCK2 could also regulate bone development via metabolic pathways. According to our results, ablation of *Pck2* brought about metabolic profile changes to E 18.5 and P0 pups, particularly in BCCAs, fatty acid synthesis, ketone body metabolism, and serine metabolism (Fig. 4e, f). Serine is generally acknowledged as an essential amino acid, which is indispensable for various physiological processes that support cell growth.^38^ Overexpression of PCK2 results in increased anabolic activity and enhanced *de novo* serine synthesis.^39^ Consistently, our data showed that serine level was decreased after *Pck2* deletion in the bone tissue, thereby validating the crucial role of PCK in serine biosynthesis. BCAAs including leucine, isoleucine, and valine have been reported to have a close relationship with insulin resistance (IR). Upregulation of circulating BCAA levels is known to be a positive marker of IR,^23,40^ which inhibits tryptophan transport into the brain across the blood–brain barrier (BBB).^41^ Interestingly, we found that *Pck2* knockout in the developing bone increased the accumulation of BCAAs at E18.5 and P0 (Fig. 4d), while it only affected tryptophan metabolism at the embryonic developmental stage (Fig. 4e). The possible reason may be that at E18.5, various important organs are in great need of tryptophan and other amino acids for rapid growth and development. Aberrant regulation of lipid metabolism results in alteration of metabolic plasticity.^42^ For example, CMPF level is drastically increased in the blood of gestational diabetes and T2DM patients, which could act directly on pancreatic beta cells. Once inside the cell, it causes deteriorated mitochondrial function, and reduces glucose-induced ATP accumulation, leading to turbulent regulation of key transcription factors and finally reducing insulin biosynthesis.^43^ Our data demonstrated that circulating CMPF, as well as other fatty acid levels could be upregulated by *Pck2* deletion in bone lysates (Fig. 4d–f), which further identified the crucial function of *Pck2* in bone metabolism.

Our in vivo data showed that *OC-Cre; Pck2*^*f/f*^ mice displayed osteoporotic symptoms including bone loss with decreased osteogenesis, as well as elevated marrow adipocyte accumulation with upregulated adipogenesis (Fig. 3), revealing the tight regulatory effect of PCK2 on bone formation. First developed for the treatment of type II diabetes, metformin was reported to promote mitochondrial respiration function via AMPK signaling.^44^ At the early stages, metformin has been found to decrease plasma glucose levels, while various functions of metformin were uncovered over time. For the intestine, metformin promotes glucose utilization and lactate production.^45^ And then, with increased energy expenditure, lactate is used to generate glucose in hepatocytes, resulting in a futile intestinal-liver cycle,^46^ which facilitates metformin’s glycemic control. Moreover, metformin has been reported to inhibit lipid secretion from intestinal epithelial cells,^47^ and promote fatty acids oxidation in adipose tissues and muscle.^48^ More recently, accumulating scientific evidence have indicated that metformin could improve the osteogenic capacity of pre-osteoblasts, MSCs, and human exfoliated deciduous teeth (SHEDs).^11,49–52^ Moreover, metformin could mitigate the detrimental effects of high glucose on osteoblast cells. Wang et al. developed a tissue-engineered construct comprised of induced pluripotent stem cell-derived mesenchymal stem cells (iPSC)-MSCs and metformin for promoting bone formation, which demonstrated an innovative role of metformin in bone tissue regeneration.^13^ As an intracellular energy sensor, AMPK signaling plays important roles in bone metabolism.^53^ Specifically, adiponectin and metformin promote the expression of osteocalcin and thus improving osteoblast differentiation upon AMPK activation, which accelerates bone formation, as well as regulating glucose metabolism. AMPK activation in osteoclast suppresses osteoclast differentiation, which suppresses bone resorption. Additionally, AMPK activation protects against oxidative stress-induced apoptosis of osteocytes, maintaining osteocyte function and bone remodeling.^54^ Metformin promotes osteoblast differentiation via AMPK activation, ERK phosphorylation, endothelial and inducible nitric oxide synthases (e/iNOS) stimulation and GSK3β/Wnt/β-catenin pathway suppression.^49^ Deficiency of epidermal growth factor receptor (*Egfr−/−* mice) shows retarded growth and severe bone defects; osteoblasts from *Egfr−/−* mice show hyperactivation of mTOR-pathway proteins, including enhanced phosphorylation of 4E-BP1 and S6, which suggests the importance of mTOR signaling in bone development.^55^ At the dose of 200 mg·kg^−1^, which was recognized as a physiological dose for rodent animal, we observed that metformin did not jeopardize bone development. However, it was reported that various concentrations of metformin make different effect on cellular functions.^56^ Therefore, we could speculate that metformin might affect development via mTOR signaling, but the dosage and duration of medication would be one of the decision factors whether the effect is positive or negative. Further study is needed to explore how clinically relevant doses of metformin inhibit mTOR and its specific effect on bone development.

Our data showed that administration of metformin significantly mitigated compromised bone development caused by *Pck2* loss via AMPK signaling (Figs. 5, 6), which also further confirmed that metformin-mediated AMPK signaling is an innovative mechanism for regulating *Pck2*-dependent bone formation.

There are some limitations in our work that need further research. Firstly, the accumulation of marrow adipocyte suggested that there might be a role of *Pck2* in regulating adipogenesis. The conditional knockout effect of *Pck2* in mesenchyme (*Prx1*) and adipose tissue in vivo could be further explored. Secondly, the specific downstream effects of metformin-AMPK signaling could be future directions for further research. Moreover, fatty acids are considered to be the one of the important fuel sources for skeletal homeostasis.^57^ In the case of fuel surplus, marrow adipocytes have been acknowledged as a large reservoir for fatty acid storage. It could also act as a source of adipokines (for example, adiponectin^58^ and RANKL^59^) which could regulate skeletal remodeling in a paracrine pattern and systemic metabolism in an endocrine manner.^60^ Therefore, it also remains a possibility *Pck2* deletion and subsequent BCAA/fatty acid accumulation in osteoblasts fuels the adipocyte formation via a paracrine manner. Consequently, CKO mice with *Prx1-Cre* and *LepR-Cre* would be constructed to evaluate the fate commitment of skeletal stem cells after *Pck2* deletion for future study.

In conclusion, this study provided further in vivo evidence that *Pck2* derived from the *OC*^*+*^ osteoblast population is prerequisite for craniofacial and long bone development. Such defects induced by *Pck2* deletion could be mitigated by metformin, at least partly via AMPK-signaling pathway. This metformin-PCK2-mediated signaling pathway could therefore be a novel mechanism for mitigating craniofacial malformation and osteoporosis, which thus opens up a new avenue for clinical treatment strategies for bone metabolic disorders.

## Materials and methods

### Mouse lines

Located on Mouse chromosome 14, The *Pck2* gene (NCBI Reference Sequence: NM_028994; Ensembl: ENSMUSG00000040618) has 10 identified exons. The ATG start codon was in exon 1 and the TGA stop codon was in exon 10 (Transcript: ENSMUST00000048781). The cKO region for conditional knockout included Exon 3~7. Deletion of this region could cause the functional deficiency of the mouse *Pck2* gene. *OC-cre* transgenic mice was purchased from Jackson Laboratories, *Pck2*^*f/f*^ and mice with a C57BL6/J background were generated by Cyagen Co., Ltd. (Suzhou, China) using a CRISPR/Cas9 based technique. This *OC-Cre* mouse model had been widely used to explore the effects of conditional gene loss in osteoblast progenitor cells on bone formation.^61^ In postnatal studies, sex-matched mice with indicated phenotypes were analyzed. Representative data from the analyses of at least three *Pck2*^*f/f*^ and *OC-Cre; Pck2*^*f/f*^ mice in each experiment are displayed in this study.

### Mice treated with metformin

Sterile saline was used for dissolving metformin hydrochloride (Sigma-Aldrich, St Louis, MO) to a final concentration of 10 mg·mL^−1^. Aliquots of 0.4 mL (4 mg) were injected intraperitoneally into mice daily, i.e., 200 mg·kg^−1^ × 0.02 kg mice. For pregnant mice, intraperitoneal injection was performed at a concentration of 200 mg·kg^−1^ every day from E18.5, and the postnatal mice were injected every other day.^2,25^ Equal volumes of sterile saline were injected to control groups. To prevent the abandonment effects of manipulation for newborns, we applied the mother’s urine to the litter after each injection.

### Whole-mount skeletal staining

Mice were harvested at E18.5 according to the standard protocol.^62^ After skinning and evisceration, the whole skeletal mount was fixed with 95% (v/v) ethanol overnight. The samples were then stained in Alcian Blue solution for 3 days (95% v/v ethanol 800 mL, acetic Acid 200 mL, alcian Blue 150 mg). Then the specimens were immersed back in in 95% ethanol for 2–5 h, followed by treatment with 2% (w/v) KOH for 24 h until mostly clear. The skeletal was then stained overnight in 1% (w/v) KOH, and 0.015% (w/v) Alizarin Red (Sigma A3757). Clear skeletons were stored in 1% (w/v) KOH, 20% (v/w) glycerol for 2 days or more. After capturing images, the skeletons were stored in a 1:1 mixture of glycerol and 95% (v/v) ethanol.

### Immunofluorescence staining of tissues

Embryos (E18.5) and early postnatal head and pups (P0 and P5) were fixed overnight in 4% (w/v) paraformaldehyde (PFA) in phosphate-buffered saline (PBS) and dehydrated according to standard protocols, for the preparation of paraffin sections (undecalcification sections). Then, the paraffin sections were permeabilized with PBT (1× PBS + 0.1% Tween 20), and were blocked in 5% (v/v) normal goat blocking serum for 20 min. For adult bone tissues (P35), samples were fixed in 4% (w/v) PFA and stored in 10% (w/v) EDTA (pH 7.4) for 4 weeks at RT (decalcification sections). After blocking, the samples from E18.5, P0, P5 and P35 were then incubated with the primary antibodies anti-Osx (ab209484, Abcam, 1:200) and anti-Opn (22952-1-AP, ZEN-BIOSCIENCE, 1: 150) overnight at 4 °C. The IF stained slides were then imaged (Beijing Genepool Biotechnology Company Limited), and analyzed by Caseviewer 2.4 software.

### Micro-computed tomography and histomorphometry analysis

For postnatal long bones, micro-CT scanning was conducted using an Inveon MM system (Siemens, Munich, Germany) as previously described.^10^ The images were analyzed using software from the manufacturer (Inveon Research Workplace; Siemens, Munich, Germany). After fixed in (w/v) 10% formalin for 24 h, femurs were under decalcification for at least 1 month. Then the femurs were dehydrated, embedded with resin, and sliced for H&E staining and IF staining. The images were analyzed with Caseviewer 2.4 software.

### Calcein-alizarin red S labeling

The dynamic bone formation was analyzed by Calcein-alizarin red S labeling. Mice (start age is P35) were administrated intraperitoneally with 20 mg·kg^−1^ calcein (Sigma, C0875-5G, 1 mg·mL^−1^ in 2% NaHCO_3_ solution) and 40 mg·kg^−1^ alizarin red S (Sigma, A5533-25G, 2 mg·mL^−1^ in H_2_O) at day 7 and day 4 before sacrifice. The tibias were fixed, dehydrated and conducted to hard tissue sections. Tibias were cut into 40-60 μm sections with a hard tissue cutting and grinding system (EXAKT Apparatebau, Hamburg, Germany). The fluorescence-labeled images were captured and analyzed using a confocal microscope (Sp8, Leica).

### Cell isolation and osteogenic induction

Primary mouse bone lysates were separated from P0 mice, followed by collagenase (type 2 and type 4, 15 mg·mL^−1^, Worthington Biochemical Corporation) digestion of the tibiae and femurs. After centrifugation, cells were cultured with MSCM medium (Science Cell), containing 500 mL of basal medium, 25 mL of fetal bovine serum (FBS, catalog number 0025), 5 mL of MSCGS (MSCGS, catalog number 7552) and 5 mL of penicillin/streptomycin solution (P/S, catalog number 0503). Cells were allowed to attach for 2–3 days. After 12–14 days of culture, the cells were passaged using trypsin (Sigma-Aldrich). When the confluence reached 80%, cells were cultured with osteogenic media (Alpha-MEM, 10% FBS, 100 U·mL^−1^ penicillin, 100 μg·mL^−1^ streptomycin, 100 μmol·L^−1^ ascorbic acid, and 10 mmol·L^−1^ β-glycerol phosphate) for the various indicated time points.^2^

### Metabolic analysis

The pup lysates of E18.5 embryos and P0 were isolated after removing both ends to exclude cartilage. The treated pups were grinded in the presence of liquid nitrogen. Three *Pck2*^*f/f*^ (Control) and *OC-Cre; Pck2*^*f/f*^ (Test) mice embryos and newborns were utilized as samples. Metabolomic analysis of *Pck2*^*f/f*^ (Control) and *OC-Cre; Pck2*^*f/f*^ (Test) mice embryos and newborn pups were carried out by Lipidall Technologies Company Limited, using Ultra-high Performance Liquid Chromatography (UPLC) and Tandem quadrupole time-of fligh (QTOF).

### RNA isolation and qRT-PCR

Total RNA was isolated from pups of E18.5 and P0 using the TRIzol reagent (Invitrogen) as previously described.^63^ Reverse transcription was carried out utilizing the *Evo M-MLV*RT Premix (Applied Biosystems, Foster City, CA, USA). Quantitative RT–PCR was conducted with SYBR Green Pro Taq HS (Accurate Biotechnology (human) Co., Ltd) and the 7500 Real-Time PCR Detection System (Applied Biosystems, Foster City, CA, USA). The primer sequences are as follows: *Gapdh*, 5’-CCACTCTTCCACCTTCG-3’ and 5’-GTGGTCCAGGGTTTCTTAC-3’; *Col1a1*, 5′-TAGGCCATTGTGTATGCAGC-3′ and 5′-ACATGTTCAGCTTTGTGGACC-3′; *Osx*, 5′-ATGGCGTCCTCTCTGCTTG-3′ and 5′-TGAAAGGTCAG CGTATGGCTT-3′; *Runx2*, 5′-TCCACSSGGACAGAGTCAGATTACAG-3′ and 5′-CAGAAGTCAGAGGTGGCAGTGTCATC-3′; *Alp*, 5′-CGGGACTGGTACTCGGATAA-3′ and 5′-ATTCCACGTCGGTTCTGTTC-3′; *Pck2*, 5′-CCCTGACTGGACATGGGGAT-3′, and 5′-GGCAAAGCACTTCTTGCCCA-3′.

### Western blot analysis

Bone samples were lysed using whole lysis buffer as previously described.^63^ Cells were lysed with radioimmunoprecipitation assay (RIPA) buffer (Beijing Huaxing Boca Genetic Technology Co., HX1862), supplemented with protein phosphatase inhibitor mixture (Beijing Huaxing Boca Genetic Technology Co., HX1864). Western blotting analyses were performed as standardized. Primary antibodies against GAPDH (1:5 000; rabbit, Beijing Huaxing Boca), PCK2 (1:1 000; rabbit, Cell Signaling Technology), p-AMPK (1:1 000; rabbit, Proteintech) and AMPK (1:1 000; rabbit, Proteintech), were utilized in this study.

### ALP Staining

After culturing in osteogenic medium (OM) for 7 days, cells were collected. Cells were cleansed with PBS three times, and then were fixed in 95% (v/v) ethanol at room temperature (RT) for 30 min. After that, the cells were incubated with a 5-bromo-4-chloro-3-indolyl phosphate4-nitro blue tetrazolium (BCIP/NBT) staining kit (CWBIO, Beijing, China) for 20 min and then washed with distilled water three times, followed by imaging under a microscope.

### Statistical analysis

The representative data in this work were mean ± SD. At least three or more independent biological repeated experiments were included for quantification. GraphPad Prism 8 (GraphPad Software) was used for analyzing data. For comparison between two groups, statistical analysis was conducted by a two-tailed Student’s *t* test to calculate significance. The *P* values less than 0.05 were identified as significant.

## Supplementary information

Metformin mitigates Pck2-mediated bone abnormity

## Data Availability

The data presented in this study are included in all the figures and supplementary materials, which could be provided from the corresponding author after reasonable demand.
