# Supplementary material for: Metformin can mitigate skeletal dysplasia caused by Pck2 deficiency
Source: Int J Oral Sci. 2022 Nov 15;14:54. doi: 10.1038/s41368-022-00204-1 (PMC9663691; doi:10.1038/s41368-022-00204-1)
Supplement: Supplementary file 1 — Metformin mitigates Pck2-mediated bone abnormity [file 41368_2022_204_MOESM1_ESM.docx]

**Supplementary data**

**Metformin can mitigate skeletal dysplasia caused by *Pck2* deficiency**

Running title: Metformin mitigates *Pck2-*mediated bone abnormity


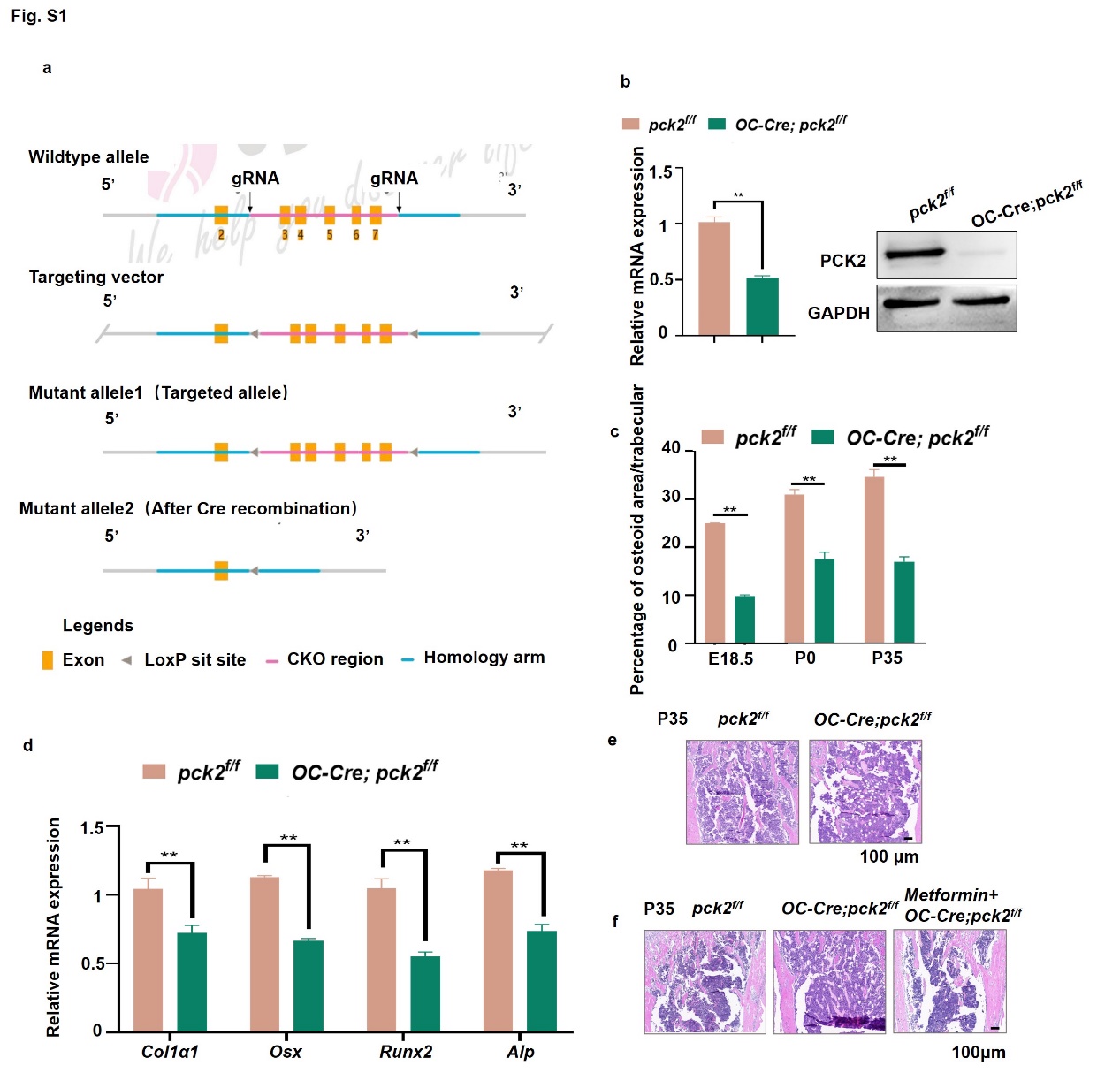


**Fig. S1** *OC-Cre; Pck2^f/f^* mice and *Pck2^f/f^* mice were successfully generated. **a** The strategy for generating the Pck2 conditional knockout Mouse model (C57BL/6N) by CRISPR/Cas-mediated genome engineering. **b** The knockout efficiency was confirmed by qRT-PCR and western blot in the lysates of pups from P0 mice with indicated genotype. ***P* < 0.01, n=3. **c** The percentage of osteoid area in femur at E18.5 and P0, and the percentage of trabecular in femur at P35. ***P* < 0.01, n=3. d The relative mRNA expression levels of *Col1a1, Osx, Runx2* and *Alp* in tissue lysates from the bone of E18.5 pups with indicated genotypes/treatments. ***P* < 0.01, n=3. **e, f** Images of adipose in the femur distal femur marrow in *OC-Cre;* *Pck2^f/f^* mice and *Pck2^f/f^* mice at P35. Scale bar: 100 μm, n=3.


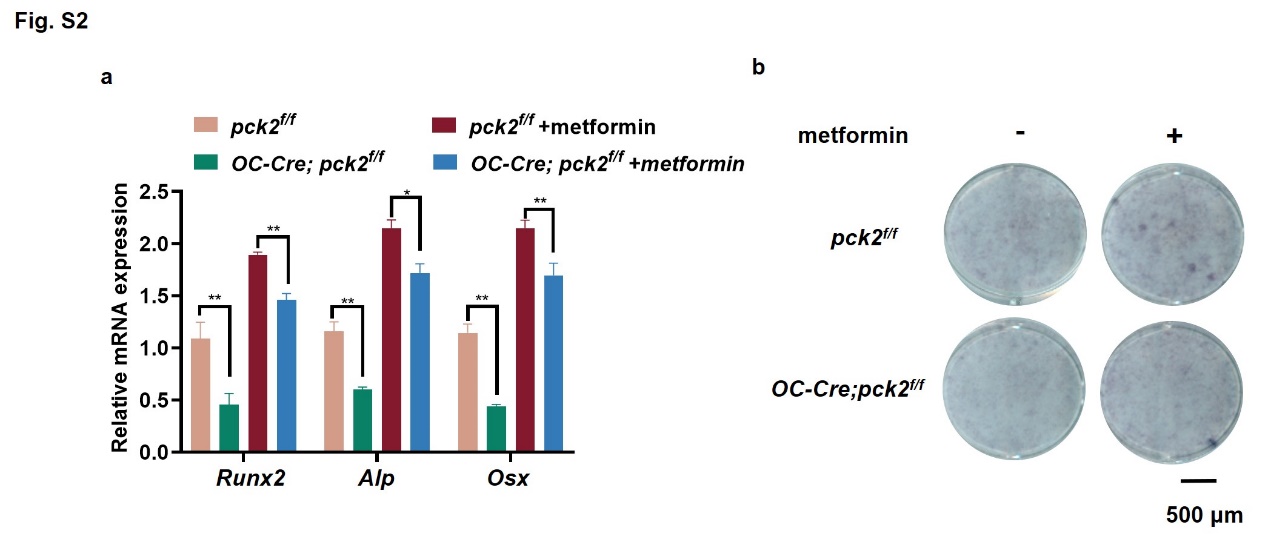


**Fig. S2** Metformin mitigated the bone phenotypes of *OC-Cre; Pck2^f/f^* mice. **a** Expression of osteogenic marker genes *Runx2, Osx* and *Alp* in the tissue lysates from the bones of P0 pups with indicated genotypes upon osteogenic induction for 7 days. ***P* < 0.01. n = 3. **b** ALP staining of mesenchymal cells from the bones of P0 pups with indicated genotypes upon osteogenic induction for 7 days. Scale bar: 500 μm, n=3.
